# Anterior Insula GABA Levels Correlate with Emotional Aspects of Empathy: A Proton Magnetic Resonance Spectroscopy Study

**DOI:** 10.1371/journal.pone.0113845

**Published:** 2014-11-24

**Authors:** Qianfeng Wang, Zhuwei Zhang, Fang Dong, Luguang Chen, Li Zheng, Xiuyan Guo, Jianqi Li

**Affiliations:** 1 Shanghai Key Laboratory of Magnetic Resonance and Department of Physics, East China Normal University, Shanghai, China; 2 School of Psychology and Cognitive Science, East China Normal University, Shanghai, China; Chiba University Center for Forensic Mental Health, Japan

## Abstract

***Background*:**

Empathy is a multidimensional construct referring to the capacity to understand and share the emotional and affective states of another person. Cerebral γ-aminobutyric acid (GABA)-ergic levels are associated with a variety of neurological and psychiatric disorders. However, the role of the GABA system in different dimensions of empathy has not been investigated.

***Materials and Methods*:**

Thirty-two right-handed healthy volunteers took part in this study. We used proton magnetic resonance spectroscopy to determine GABA concentrations in the anterior insula (AI) and the anterior cingulate cortex (ACC) and to examine the relationship between the GABA concentrations and the subcomponents of empathy evaluated by the Interpersonal Reactivity Index (IRI).

***Result*:**

Pearson correlation analyses (two-tailed) showed that AI GABA was significantly associated with the empathy concern score (r = 0.584, p<0.05) and the personal distress score (r = 0.538, p<0.05) but not significantly associated with other empathy subscales. No significant correlation was found between ACC GABA and empathy subscores.

***Conclusion*:**

Left AI GABA was positively correlated with the emotional aspects of empathy. These preliminary findings call into question whether AI GABA alterations might predict empathy dysfunction in major psychiatric disorders such as autism and schizophrenia, which have been described as deficits in emotional empathic abilities.

## Introduction

Empathy is a multidimensional construct and usually refers to the capacity to understand and share the emotional and affective states of another person. Empathy is believed to be important in social behaviors [1]. Empathy dysfunction has been viewed as the central characteristic of psychopathological syndromes in severe mental disorders such as schizophrenia and autism [2]–[5]. A large body of neuroimaging and neurophysiology studies have focused on empathy for pain, demonstrating that the perception of other's pain activates similar regions of the brain, including the bilateral anterior insula (AI), and the anterior cingulate cortex (ACC) [6]–[12].

As the primary neurotransmitters in the human brain, glutamate (Glu) and γ-aminobutyric acid (GABA)-ergic mechanisms are important for a variety of neurological and psychiatric disorders, including Parkinson's disease, and Alzheimer's dementia [13]–[16]. Studies on various regions of the brain have revealed links between empathy and neurotransmitters in humans. Montag et al [17] applied proton magnetic resonance spectroscopy (^1^H-MRS) to measure the absolute concentrations of cerebral glutamate in the ACC, left dorsolateral prefrontal (PFC) and left hippocampus and found that dorsolateral prefrontal cortex (DLPFC) glutamate levels showed a significant negative correlation with the “perspective taking” score. GABA, the main inhibitory transmitter in the brain, has been shown in a number of studies to be correlated with various disorders such as autism [18]. More recently, Rosso et al [19] reported that the anterior insula GABA was significantly lower in posttraumatic stress disorder (PTSD) subjects than in controls, although dorsal ACC GABA did not differ significantly. In studies of panic disorder, GABA was significantly reduced in the ACC [20], [21].

As mentioned above, the AI and the ACC have been identified as key regions involved in empathy for pain. However, to our knowledge, the role of the GABA system for different dimensions of empathy has not been investigated thus far, and the relationship between the GABA system and empathy remains unclear. In this study of the regional brain using ^1^H-MRS to detect GABA, the AI and the ACC were chosen as regions of interest due to their involvement in empathy. Specifically, in the present study, ^1^H-MRS was used to measure the concentrations of cerebral GABA in the AI and the ACC at rest and to examine the relationship between the GABA concentrations and the subcomponents of empathy evaluated by Interpersonal Reactivity Index (IRI) [22]. It was hypothesized that the GABA concentrations in the AI and/or the ACC would be correlated with subcomponents of empathy.

## Materials and Methods

### Participants

Thirty-two right-handed volunteers (eleven females, mean age = 25.2 years, *SD* = 1.8, range = 22–30 years) were recruited from the East China Normal University. None of the subjects had a history of neurological or psychological disorders. All participants gave written informed consent before scanning. The study was approved by the Ethics Committee of the East China Normal University.

### MRS acquisition and analysis

**Figure 1:**
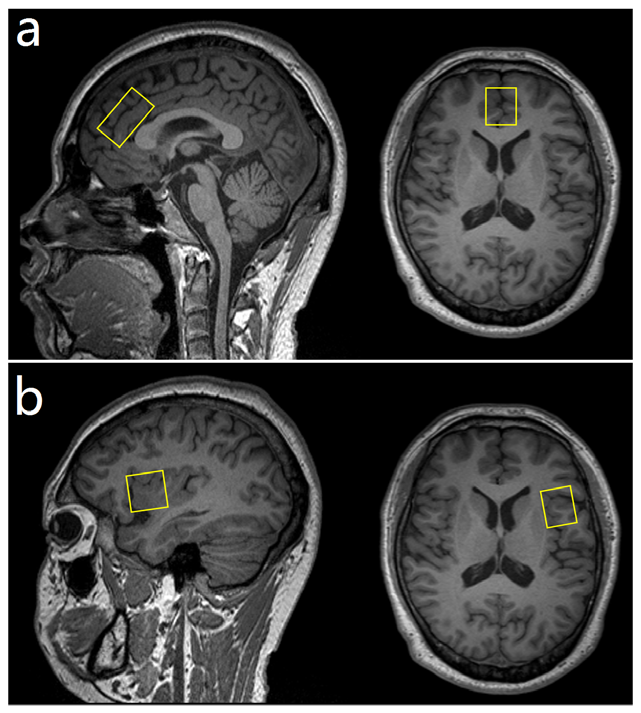
*T*_1_-weighted images depicting voxel placement in the ACC (a) and the left AI (b) of a representative participant. Voxel dimensions: ACC, 20 mm (RL)×18 mm (AP)×30 mm (FH); AI, 20 mm (RL)×25 mm (AP)×25 mm (FH).

All measurements were carried out on a 3T whole-body MR scanner (Magnetom Trio TIM, Siemens Medical Solutions, Erlangen, Germany) using a twelve-channel, phased array, receive-only head matrix coil for signal detection. Before MRS acquisition, a three-dimensional high-resolution T_1_-weighted image was acquired to guide voxel placement (MPRAGE; FOV = 240 mm×240 mm, TR = 1900 ms, TI = 900 ms, TE = 2.46 ms, 192 slices, spatial resolution = 1×1×1 mm^3^, flip angle = 9 degrees). Single voxel edited ^1^H-MR Spectra were acquired at rest from two volumes of interest (VOI) in each subject. One VOI of 20×18×30 mm^3^ = 10.8 ml was placed in the ACC (Figure 1a), while a second one (20×25×25 mm^3^ = 12.5 ml) was located in the left AI (Figure 1b). A single-volume, MEGA point-resolved spectroscopy (PRESS), J-difference editing sequence was used to measure total GABA [23]. Spectral editing was achieved using a 12.8 ms, dual-banded, Gaussian inversion pulse with a water suppression band at 4.7 ppm and an editing band alternating between 1.9 ppm (edit on) and 7.5 ppm (edit off) in even and odd acquisitions, respectively. For each spectrum, 196 spectral averages were acquired with a repetition time of 2000 ms and an echo time of 68 ms [24], [25], resulting in an acquisition time of approximately 6.5 min.

All spectra were analyzed using jMRUI software [26]. Prior to signal averaging, the phase and frequency of each individual spectrum were corrected to account for phase instabilities and frequency drifts, respectively. Phase adjustment was performed based on the location of the residual water peak and frequency alignment was performed using the location of the creatine peak [25], [27], [28]. The difference spectra were then obtained by subtracting the signals from alternate scans with the selective pulse applied at 1.9 ppm (edit on) and 7.5 ppm (edit off). NAA can be quantified directly from the edited difference spectrum. Hence, GABA concentration was used in subsequent steps as a ratio to NAA. Because measurements of GABA using the MEGA-PRESS sequence contain some contribution from co-edited macromolecule signals [27], [29], the notation GABA+ was used to indicate that GABA plus macromolecule measurements. The ratio of GABA+ to NAA was determined using the AMARES package [30] that is integrated within the jMRUI software [26]. Prior to fitting in jMRUI, all spectra were apodized with a 4 Hz Gaussian filter, and residual water peak was removed using a Hankel Lanczos singular value decomposition (HLSVD) filter. NAA was modelled as a single, inverted Lorentzian peak. GABA+ was modelled as a pair of Lorentzian peaks with the same line width as NAA, and the phase was set to +180° relative to the inverted NAA signal [27].

The signal-to-noise ratio (SNR) was calculated using the difference spectrum following phase adjustment such that the NAA peak was upright with a phase of 0 degrees for each subject [31]. Signal was calculated as the maximum intensity of the real part of the NAA peak in the phased difference spectrum, noise was calculated as the standard deviation of the real part of the noise in a signal-free part of the spectrum [31]. SNRs for ACC and AI lower than 90 were excluded from the further analyses. The mean SNRs in ACC and AI were 166 and 144, respectively. Creatine line-width and fit uncertainty (SD/amplitude ratio) were obtained from the summed spectra [31]. Any spectra were rejected as being of insufficient quality if they showed line broadening (line-width >8 Hz) or had high fit uncertainty of the Creatine peak in AMARES (SD/amplitude ratio >0.20) or had low SNR (SNR<90). All insufficient quality datasets were excluded. For the remaining datasets, the mean line width and SD/amplitude ratio in ACC are 6.6 Hz and 0.115, respectively, the mean line width and SD/amplitude ratio in AI are 6.2 Hz and 0.096, respectively. Independent sample t-test were performed to analyze group differences between these subjects. Bonferroni corrections were used to counteract the problem of multiple comparisons (six correlation analyses). Within-group comparisons were performed using paired t-tests. All tests were performed at a two-tailed level of significance of 5%.

### Assessment of empathic abilities

The subjects' empathic abilities were evaluated using a multidimensional questionnaire, the Interpersonal Reactivity Index (IRI), which has been widely used to assess empathy ability [22], [32]. The IRI assesses four dimensions of empathic responses on a 5-point Likert scale ranging from 0 (never) to 4 (always). ‘Perspective taking’ (PT) assesses the tendency to spontaneously adopt the psychological point of view of others and to reason about their mental states. The subscale ‘fantasy’ (FS) measures the tendency to identify with fictitious characters in books and movies. The ‘empathic concern’ (EC) scale measures respondents' prosocial feelings of warmth, compassion and concern for others. ‘Personal distress’ (PD) measures the personal feelings of anxiety and discomfort in response to the distress of others. Two subscales were designed to measure the cognitive elements of empathy: PT and FS. The other two subscales were designed to measure the emotional aspects of empathy: EC and PD. Higher subscale scores represent higher empathic tendencies [4], [22], [32], [33].

Participants were asked to complete the IRI independently after MRS data acquisition. Statistical calculations were carried out using SPSS 16.0 (SPSS, Chicago, Illinois) for Windows.

## Results

All 32 subjects underwent MRS measurements as scheduled. Nine subjects whose spectra were acquired in the AI and five subjects whose spectra were acquired in the ACC were excluded due to non-valid (insufficient quality) MRS data. The MRS data were analyzed for 23 subjects (nine females, mean age = 25.3, *SD* = 1.9, range = 22–30) in AI group and 27 subjects (eleven females, mean age = 25.1, *SD* = 1.8, range = 22–30) in ACC group.

**Figure 2:**
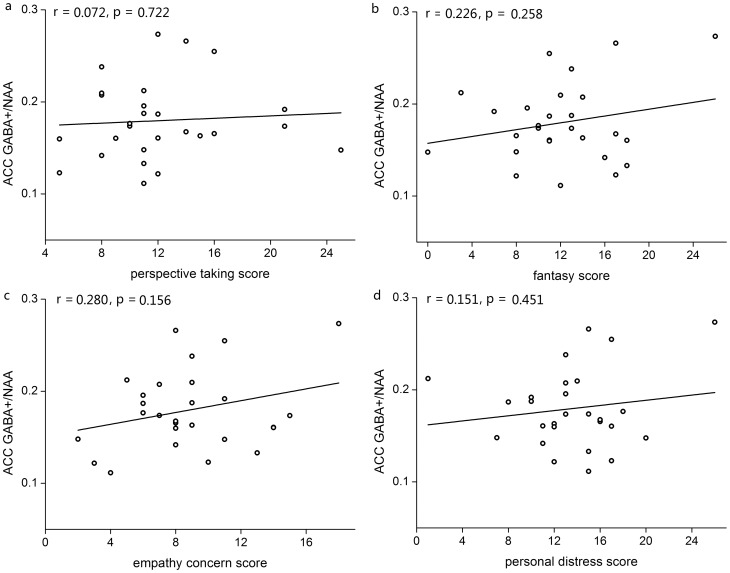
Correlations (Pearson) between AI GABA levels (relative to NAA) and empathy subscores: a, perspective taking; b, fantasy score; c, empathy concern score; and d, personal distress score. Significant positive correlations were observed in the EC score and the PD score.

**Figure 3:**
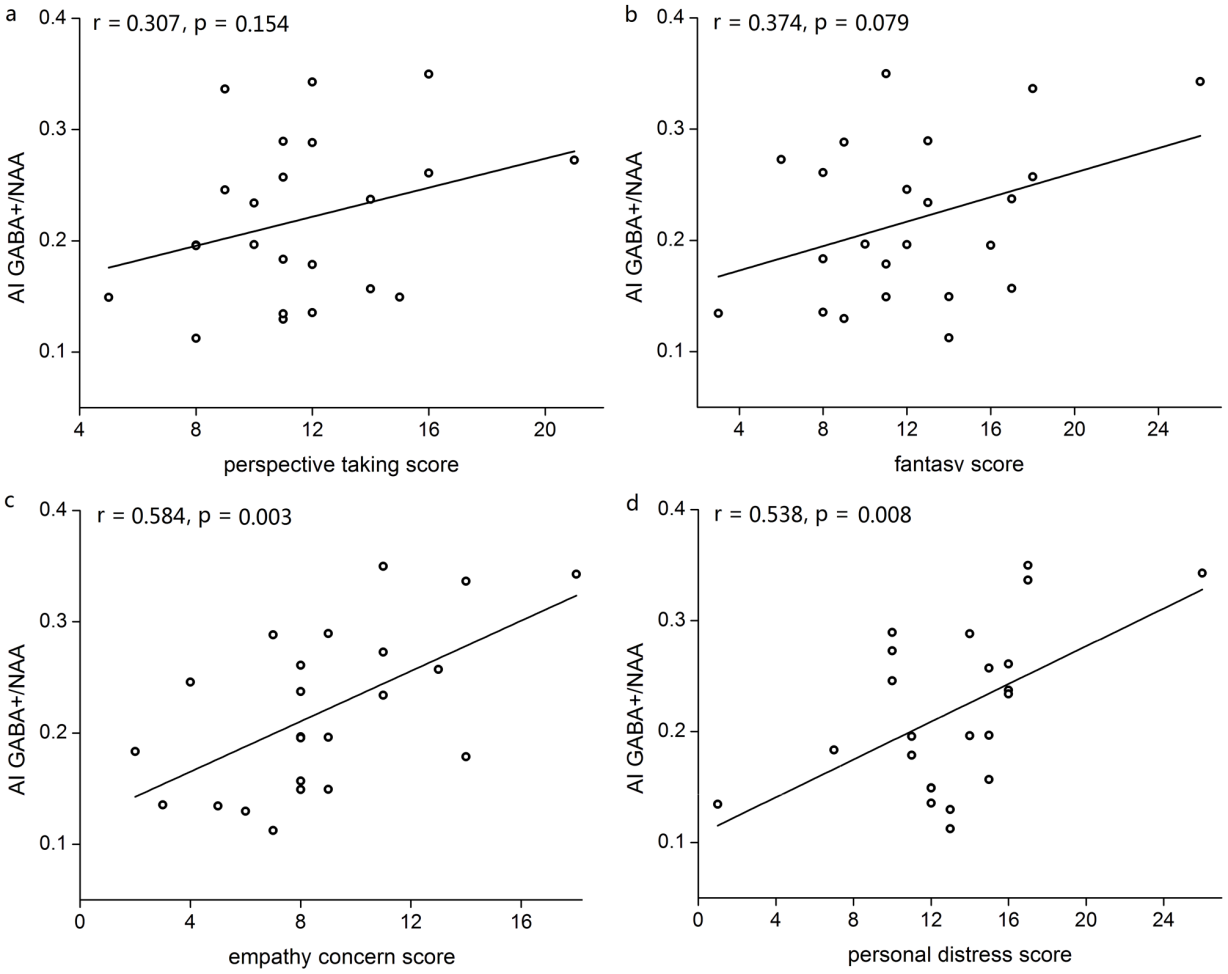
Correlations (Pearson) between ACC GABA levels (relative to NAA) and empathy subscores: a, perspective taking; b, fantasy score; c, empathy concern score; and d, personal distress score.

The mean values of GABA+ concentration (relative to NAA) in the AI of 23 subjects and in the ACC of 27 subjects, as well as mean IRI subscores, are presented in Table 1. Pearson correlation analyses (two-tailed) showed that the GABA+ concentration in the AI was significantly associated with the empathy concern score (r = 0.584, p<0.05, with 95% confidence interval of [0.226, 0.803]) and the personal distress score (r = 0.538, p<0.05, with 95% confidence interval of [0.162, 0.778]) but not significantly associated with the perspective taking score (r = 0.307, p>0.05) or the fantasy score (r = 0.374, p>0.05) (Figure 2). Analyses of GABA+ concentration in the ACC and empathy subscales did not reveal significant correlations (all ps>0.05) (indicated in Figure 3). There were no effects of gender (T = 0.284, df = 25, p>0.05 for ACC; T = 0.987, df = 21, p>0.05 for AI) and age (T = 0.700, df = 25, p>0.05 for ACC; T = 0.874, df = 21, p>0.05 for AI) on GABA concentrations. There was no significant difference between the two sub-regions for mean GABA concentrations (T = 1.594, df = 18, p>0.05) for the identical subgroup. There was no any relationship between the MRS data quality (line-width, SNR) and the empathy scales (all ps>0.05).

**Table 1:** Mean values of GABA+ concentration and IRI subscales.

	GABA+/NAA	PT	FS	EC	PD
AI (n = 23)	0.22±0.08	11.57±3.40	12.35±4.91	8.74±3.73	13.17±4.57
ACC (n = 27)	0.18±0.04	12.11±4.66	12.07±5.19	8.85±3.73	13.59±4.60

(PT = perspective taking; FS = fantasy score; EC = empathy scale; PD = personal distress; AI = anterior insula; ACC = anterior cingulate cortex). The uncertainties indicate standard deviation.

## Discussion

To our knowledge, this is the first ^1^H-MRS study reporting associations between the subcomponents of empathy and regional brain GABA+ concentrations in the AI and ACC. The main findings in this preliminary study were that there were significant positive correlations of the AI GABA+ concentrations with the two emotional empathy scales (empathy concern and personal distress). No associations were found with regard to the other subscales of the IRI. No significant relationship was found between the ACC GABA+ concentrations and empathy subscales.

The GABA system is the major inhibitory system in the human brain [13]. The strongest evidence suggests that GABA is associated with a variety of neurological and psychiatric disorders [13], [16], [34]. Sanacora et al [35] found that depressed patients demonstrated a highly significant reduction in occipital cortex GABA levels compared with healthy subjects.

Empathy is a complex form of psychological inference in which observation, memory, knowledge, and reasoning are combined to yield insight into the thoughts and feelings of others [36], [37]. Observing the emotional state of another person may result in the experience of similar emotions [38]. Pain is a special psychological state with great evolutionary significance that can be experienced by the individual and perceived in others [39]. The ability to experience another's pain is characteristic of empathy [12]. Neuroimaging and neurophysiological studies indicate a crucial role of the AI and the ACC involvement in empathy for pain. The insula subserves affective/interoceptive awareness, i.e., the integration of bodily sensations and cognitive, evaluative processes [40], it has been proposed to be relevant for emotion processing [41] and incidental self-processing of negative information [42]. Wiebking et al [43] demonstrated that the GABA+/NAA concentrations in the left insula are particularly associated with neural activity during interoceptive awareness compared to exteroceptive awareness. Singer et al [12] found that the activation levels of the left insula and the ACC during empathy-related conditions (pain vs. no pain) are significantly correlated with individual differences in empathy as measured by the empathic concern scale. Fan et al [44] reported that the left anterior insula was active in both forms of empathy. In the current study, we found that the AI GABA+ concentration was associated with the empathy concern scale as well as the personal distress scale, suggesting that the cerebral GABA system might be involved in empathy. This finding is compatible with previous studies showing a role of the AI in the pain empathy. The ACC is part of a neural circuit regulating cognitive and emotional processing and is regarded as one of the critical structures for empathetic pain processing [20]. Recently, Ernst et al [45] found that GABA concentrations in ACC were selectively associated with alexithymia. However, in this study, no correlation was found between the ACC and empathy subscales.

A considerable neuroimaging studies have convincingly demonstrated that anterior insula cortex and anterior cingulate cortex are both activated during empathic pain processing. However, Gu et al [46] showed that only anterior insula cortex, but not anterior consulate cortex, lesions resulted in prominent deficits in both explicit and implicit empathic pain perception. The current study revealed that left AI but not ACC GABA was positively correlated with the emotional aspects of empathy, suggesting there are clear distinctions between the roles of the AI and ACC. It was noting that the MRS voxel was located more anterior in the regions of ACC. Further studies could assess the relationship between GABA concentrations in the dACC-aMCC and empathy.

Studies have suggested that women may be more empathetic than men [20], [47], furthermore, there is evidence for age-related changes in empathy [48]. The limited sample size did not allow a more in-depth analysis of gender and age-related differences. Further research on the association between GABA and the empathy subscale may focus on assessing gender and age-related differences.

In this study, we sampled MR spectra only in the ACC and the AI. An fMRI study [49] showed a significant association between the empathic concern scale (EC) scores of the IRI and activity in the inferior frontal gyrus (IFG) while watching action sequences. Gazzola et al [50] found that individuals who scored higher on the perspective taking (PT) scale activated the mirror neuron system (MNS) more strongly. Montag et al [17] reported that prefrontal cortex glutamate levels correlate with the PT score. Further studies could also assess the relationship between GABA concentrations in these brain regions and empathy. Our study has other limitations. Near et al [51] suggested that accurate quantification of GABA using MEGA-PRESS is complicated by spectral co-editing of macromolecular resonances. The spectrum dataset of the current study may be contaminated by co-edited macromolecules and other unwanted contaminants for short editing pulse. In addition, the current study didn't take tissue fraction into account for analyses, which is another limitation. Future study may include tissue proportions as covariates for further analyses.

In summary, ^1^H-MRS GABA in the left AI was positively correlated with the emotional facets of empathy subscales of the IRI. This may be consistent with well-replicated evidence of insula activity in the functional magnetic imaging studies of empathy for pain. Our findings call into question whether AI GABA alterations might predict dysfunction of empathy in major psychiatric disorders such as autism and schizophrenia, which have been suggested to involve deficits in emotional empathic abilities.

## Supporting Information

File S1 Table S1, GABA quantification in ACC and IRI subscores. Table S2, GABA quantification in AI and IRI subscores.
(DOCX)
