# Supplementary material for: Anterior Insula GABA Levels Correlate with Emotional Aspects of Empathy: A Proton Magnetic Resonance Spectroscopy Study
Source: PLoS One. 2014 Nov 24;9(11):e113845. doi: 10.1371/journal.pone.0113845 (PMC4242717; doi:10.1371/journal.pone.0113845)
Supplement: File S1 — Table S1, GABA quantification in ACC and IRI subscores. Table S2, GABA quantification in AI and IRI subscores. (DOCX) [file pone.0113845.s001.docx]

**Table S1.** GABA quantification in ACC and IRI subscores.

| **Subject** | **gender** | **ages** | **PT** | **FS** | **EC** | **PD** | **ACC GABA+/NAA** | **Fit uncertainty** | **LW** | **SNR** |
| --- | --- | --- | --- | --- | --- | --- | --- | --- | --- | --- |
| 1 | f | 25 | 11 | 13 | 9 | 10 | 0.187 | 0.098 | 7.03 | 167.093 |
| 2 | m | 28 | 11 | 9 | 6 | 13 | 0.196 | 0.126 | 7.35 | 152.586 |
| 3 | m | 24 | 14 | 17 | 8 | 15 | 0.266 | 0.133 | 7.23 | 223.842 |
| 4 | m | 24 | 8 | 14 | 7 | 13 | 0.207 | 0.108 | 5.86 | 114.765 |
| 5 | f | 30 | 11 | 3 | 5 | 1 | 0.212 | 0.125 | 7.03 | 180.695 |
| 6 | m | 25 | 12 | 11 | 14 | 11 | 0.161 | 0.106 | 5.86 | 156.900 |
| 7 | m | 27 | 15 | 14 | 9 | 12 | 0.163 | 0.108 | 7.03 | 146.252 |
| 8 | m | 25 | 16 | 11 | 11 | 17 | 0.255 | 0.130 | 7.21 | 212.341 |
| 9 | m | 24 | 10 | 10 | 7 | 15 | 0.174 | 0.094 | 5.86 | 120.366 |
| 10 | f | 23 | 8 | 13 | 9 | 13 | 0.238 | 0.129 | 7.69 | 162.487 |
| 11 | m | 23 | 12 | 26 | 18 | 26 | 0.273 | 0.114 | 7.9 | 163.192 |
| 12 | f | 23 | 8 | 12 | 9 | 14 | 0.210 | 0.117 | 6.72 | 136.438 |
| 13 | m | 25 | 10 | 10 | 6 | 18 | 0.177 | 0.117 | 7.4 | 264.946 |
| 14 | f | 25 | 21 | 6 | 11 | 10 | 0.192 | 0.145 | 7.03 | 185.027 |
| 15 | m | 27 | 16 | 8 | 8 | 16 | 0.166 | 0.078 | 4.69 | 206.358 |
| 16 | f | 25 | 5 | 11 | 8 | 12 | 0.160 | 0.136 | 5.2 | 142.427 |
| 17 | m | 24 | 11 | 18 | 13 | 15 | 0.133 | 0.132 | 7.69 | 166.236 |
| 18 | m | 28 | 11 | 12 | 4 | 15 | 0.111 | 0.135 | 7.03 | 194.350 |
| 19 | f | 25 | 12 | 8 | 3 | 12 | 0.122 | 0.082 | 4.51 | 197.851 |
| 20 | m | 27 | 21 | 13 | 15 | 13 | 0.173 | 0.126 | 7.03 | 142.570 |
| 21 | f | 22 | 11 | 8 | 2 | 7 | 0.148 | 0.067 | 4.69 | 186.160 |
| 22 | f | 24 | 12 | 11 | 6 | 8 | 0.187 | 0.127 | 7.23 | 124.344 |
| 23 | m | 27 | 14 | 17 | 8 | 16 | 0.168 | 0.086 | 5.86 | 204.975 |
| 24 | m | 25 | 9 | 18 | 14 | 17 | 0.161 | 0.076 | 7.63 | 204.114 |
| 25 | f | 24 | 25 | 0 | 11 | 20 | 0.148 | 0.121 | 5.86 | 99.890 |
| 26 | f | 24 | 8 | 16 | 8 | 11 | 0.142 | 0.135 | 7.03 | 102.633 |
| 27 | m | 25 | 5 | 17 | 10 | 17 | 0.123 | 0.125 | 7.04 | 125.450 |

**Table S2.** GABA quantification in AI and IRI subscores.

| **Subject** | **gender** | **ages** | **PT** | **FS** | **EC** | **PD** | **AI GABA+/NAA** | **Fit uncertainty** | **LW** | **SNR** |
| --- | --- | --- | --- | --- | --- | --- | --- | --- | --- | --- |
| 1 | m | 25 | 9 | 18 | 14 | 17 | 0.336 | 0.101 | 4.69 | 134.938 |
| 2 | m | 27 | 14 | 17 | 8 | 16 | 0.237 | 0.137 | 7.69 | 114.186 |
| 3 | m | 23 | 12 | 26 | 18 | 26 | 0.343 | 0.095 | 5.86 | 114.530 |
| 4 | m | 25 | 16 | 11 | 11 | 17 | 0.350 | 0.080 | 7.03 | 137.885 |
| 5 | m | 27 | 10 | 13 | 11 | 16 | 0.234 | 0.116 | 6.43 | 139.315 |
| 6 | f | 26 | 11 | 13 | 9 | 10 | 0.290 | 0.102 | 7.69 | 112.815 |
| 7 | m | 24 | 11 | 18 | 13 | 15 | 0.257 | 0.089 | 4.68 | 135.003 |
| 8 | m | 27 | 16 | 8 | 8 | 16 | 0.261 | 0.079 | 4.83 | 160.255 |
| 9 | f | 25 | 21 | 6 | 11 | 10 | 0.273 | 0.076 | 6.13 | 114.057 |
| 10 | f | 23 | 9 | 12 | 4 | 10 | 0.246 | 0.094 | 5.86 | 166.925 |
| 11 | m | 28 | 12 | 9 | 7 | 14 | 0.288 | 0.083 | 7.03 | 94.187 |
| 12 | m | 24 | 8 | 14 | 7 | 13 | 0.112 | 0.119 | 7.69 | 152.695 |
| 13 | f | 23 | 8 | 12 | 9 | 14 | 0.196 | 0.090 | 6.43 | 114.334 |
| 14 | m | 25 | 12 | 11 | 14 | 11 | 0.179 | 0.099 | 5.86 | 115.633 |
| 15 | f | 25 | 12 | 8 | 3 | 12 | 0.135 | 0.085 | 5.86 | 170.975 |
| 16 | m | 24 | 14 | 17 | 8 | 15 | 0.157 | 0.092 | 7.03 | 288.437 |
| 17 | m | 27 | 15 | 14 | 9 | 12 | 0.149 | 0.095 | 7.69 | 152.671 |
| 18 | m | 25 | 10 | 10 | 8 | 15 | 0.197 | 0.105 | 5.86 | 114.802 |
| 19 | f | 22 | 11 | 8 | 2 | 7 | 0.183 | 0.077 | 4.83 | 134.204 |
| 20 | m | 28 | 11 | 9 | 6 | 13 | 0.130 | 0.081 | 4.69 | 233.863 |
| 21 | f | 30 | 11 | 3 | 5 | 1 | 0.134 | 0.078 | 7.03 | 207.683 |
| 22 | f | 24 | 8 | 16 | 8 | 11 | 0.196 | 0.129 | 7.69 | 102.354 |
| 23 | f | 25 | 5 | 11 | 8 | 12 | 0.149 | 0.107 | 4.68 | 104.526 |
